# Challenging the role of social norms regarding body weight as an explanation for weight, height, and BMI misreporting biases: Development and application of a new approach to examining misreporting and misclassification bias in surveys

**DOI:** 10.1186/1471-2458-11-331

**Published:** 2011-05-18

**Authors:** Jonathan R Brestoff, Ivan J Perry, Jan Van den Broeck

**Affiliations:** 1 Department of Epidemiology and Public Health & HRB Centre for Health and Diet Research, University College Cork, Ireland

**Keywords:** Social norms, social desirability, BMI bias, misreporting bias, weight bias, height bias, misclassification bias, survey

## Abstract

**Background:**

Cultural pressures to be thin and tall are postulated to cause people to misreport their body weight and height towards more socially normative (i.e., desirable) values, but a paucity of direct evidence supports this idea. We developed a novel non-linear approach to examining weight, height, and BMI misreporting biases and used this approach to examine the association between socially non-normative weight and misreporting biases in adults.

**Methods:**

The Survey of Lifestyles, Attitudes, and Nutrition 2007 (SLÁN 2007), a nationally representative survey of the Republic of Ireland (N = 1942 analyzed) was used. Self-reported weight (height) was classified as under-reported by ≥2.0 kg (2.0 cm), over-reported by ≥2.0 kg (2.0 cm), or accurately reported within 2.0 kg (2.0 cm) to account for technical errors of measurement and short-term fluctuations in measured weight (height). A simulation strategy was used to define self-report-based BMI as under-estimated by more than 1.40 kg/m^2^, over-estimated by more than 1.40 kg/m^2^, or accurately estimated within 1.40 kg/m^2^. Patterns of biases in self-reported weight, height, and BMI were explored. Logistic regression was used to identify factors associated with mis-estimated BMI and to calculate adjusted odds ratios (AOR) and 99% confidence intervals (99%CI).

**Results:**

The patterns of bias contributing the most to BMI mis-estimation were consistently, in decreasing order of influence, (1) under-reported weight combined with over-reported height, (2) under-reported weight with accurately reported height, and (3) accurately reported weight with over-reported height. Average bias in self-report-based BMI was -1.34 kg/m^2 ^overall and -0.49, -1.33, and -2.66 kg/m^2 ^in normal, overweight, and obese categories, respectively. Despite the increasing degree of bias with progressively higher BMI categories, persons describing themselves as too heavy were, within any given BMI category, less likely to have under-estimated BMI (AOR 0.5, 99%CI: 0.3-0.8, P < 0.001), to be misclassified in a lower BMI category (AOR 0.3, 99%CI: 0.2-0.5, P < 0.001), to under-report weight (AOR 0.5, 99%CI: 0.3-0.7, P < 0.001), and to over-report height (OR 0.7, 99%CI: 0.6-1.0, P = 0.007).

**Conclusions:**

A novel non-linear approach to examining weight, height, and BMI misreporting biases was developed. Perceiving oneself as too heavy appears to reduce rather than exacerbate weight, height, and BMI misreporting biases.

## Background

One of the most commonly used proxy measures of obesity in large cross-sectional surveys is elevated body mass index (BMI). A cost-effective, practical approach for obtaining BMI values in large numbers of individuals is to collect self-reported weight and height data, but these parameters are liable to response and recall bias. Many studies in adults have indicated that self-reported weight and height tend to be under- and over-reported, respectively, in surveys in the United States [1-4], England [5,6], Germany [7], France [8], Spain [9], Italy [10], Sweden [11,12], Finland [13], New Zealand [14], the United Kingdom [15], and Ireland [16].

It has been postulated that cultural pressures to be thin and tall cause people to intentionally or unintentionally misreport their body weight and height towards more socially normative (i.e., desirable) values [15,17,18]. This theory, proposed by Zeibland et al. (1996), has been supported mainly by indirect evidence. In particular, numerous studies show that self-report-based BMI estimates from overweight and obese individuals tend towards the normal range, thereby resulting in substantial BMI category misclassification [2,5,8,10,11,13,18,19]. Consistent with these observations, heavier individuals tend to under-report weight to greater degrees than lighter individuals [6,12,15,20], and the sensitivities of self-reported weight and self-report-based BMI categorization decrease as measured BMI category increases [2,11,16].

Three studies have directly examined the relationship between social desirability and biases in self-reported weight and height in adults, and the findings from these studies are conflicting [4,17,18]. In one small study of 56 non-obese individuals, stronger desires to conform to social norms, as measured using the Marlowe Crowne Social Desirability Scale, correlated with greater magnitudes of self-reported weight bias in females [17]. However, in another much larger study, the degree of bias in self-reported weight was negatively related to the difference between measured body weight and "socially ideal" body weight, defined as the mean self-reported weight of the sample [18]. Consistent with the second study, a third reported that those who consider themselves to be too heavy are half as likely to have a discrepancy between self-reported and measured weight than those who consider themselves to be about the right weight [4]. The association in this latter study, however, likely suffers from significant bias because the questionnaire did not reference the participants to an appropriate comparison group (e.g., those of the same age), a factor that is known to influence what constitutes a social norm [21,22].

Collectively, the evidence supporting the influence of social desirability on misreporting biases of anthropomorphic parameters is limited and controversial. Considering that social desirability and self-reported weight bias seem to be related non-linearly [18], further examination of this topic would benefit from a non-linear methodology, of which only one has been reported in the context of self-reported weight bias [4]. The current methodology does not, however, take into account technical errors of measurement (TEM) and short-term variations in weight or height, nor does it enable the examination of biases in self-report-based BMI, a parameter that better approximates adiposity. Therefore, the purposes of the present study were (1) to develop a non-linear approach to examining biased estimates of self-report-based BMI that accounts for TEM and short-term variations in weight and height; (2) to use this approach to characterize patterns of self-reported weight, height, and BMI biases in the Republic of Ireland using a nationally representative survey; and (3) to test directly the hypothesis that considering oneself to be heavier than the socially normative weight, while taking into consideration one's age and height, is positively associated with biases in self-report-based estimates of BMI.

## Methods

### Setting and population

The Survey of Lifestyle, Attitudes and Nutrition (SLÁN) 2007 is a nationally representative cross-sectional study of the adult (18+ years) population residing in the Republic of Ireland. Detailed methodology is found in the SLÁN 2007 Main Report [23]. In brief, trained interviewers conducted face-to-face interviews with 10,364 subjects selected using a multistage area probability sampling procedure. In addition, SLÁN 2007 incorporated two sub-studies for (1) the measurement of anthropomorphic characteristics (N = 967, age 18-44 years) and (2) physical examination with clinical laboratory tests (N = 1207, age 45+ years). In the main survey, self-reported height and weight were obtained and used to calculate self-report-based BMI (BMI_SR_), and in both sub-studies trained interviewers measured body height and weight for the calculation of measured BMI (BMI_M_).

### Inclusion criteria

Subjects who completed the SLÁN 2007 main survey and one of its sub-studies were selected for the present analyses, resulting in an initial sample size of 2174 (age 18+ years). Subjects were excluded if the interviewer deemed the height or weight measurements to be unreliable or slightly unreliable (N = 18) or if data was missing for BMI_M _(N = 3), self-reported height (N = 75), self-reported weight (N = 48), or any of the analysis variables (N = 68). The differences between self-reported and measured height or weight were determined, and the five most extreme cases in either direction were excluded because these cases were considered to have a high probability of data recording or entry error, as opposed to reporting error (N = 20). The total number of cases excluded was 232, resulting in a final sample size of 1942.

### Definitions of under- and over-reported weight, height, and BMI

Self-reported weight or height was deemed acceptably accurate if within ±2.0 kg or ±2.0 cm of measured weight or height, respectively. These cut-off points were determined to allow for various factors that might explain differences between self-reported and measured height and weight that can occur even in the absence of any intentional or unintentional misreporting. One factor is the technical error of measurement of weight (negligible) and height (0.5 cm), and another is temporal variation in weight and height depending on food/liquid consumption, water balance, and postural differences. In addition, height and weight in an individual can vary with age, and subjects may have meticulously reported their weight and height based on a measurement that was taken months or years ago. Finally, most subjects (97%) reported their height in terms of feet and inches, which entails additional errors. The combined effects of errors in self-reported height and weight on BMI bias were simulated and led us to the conclusion that self-reported BMI should be considered accurate if within ±1.40 kg/m^2^, under-estimated if < -1.40 kg/m^2^, or over-estimated if > +1.40 kg/m^2 ^(see additional file 1: Method of BMI error simulation to establish a BMI accuracy definition). This simulation method can be readily applied in samples of other populations, as it uses the mean measured weight and height of the sample.

### Patterns of bias in height, weight, and BMI

**Figure 1:**
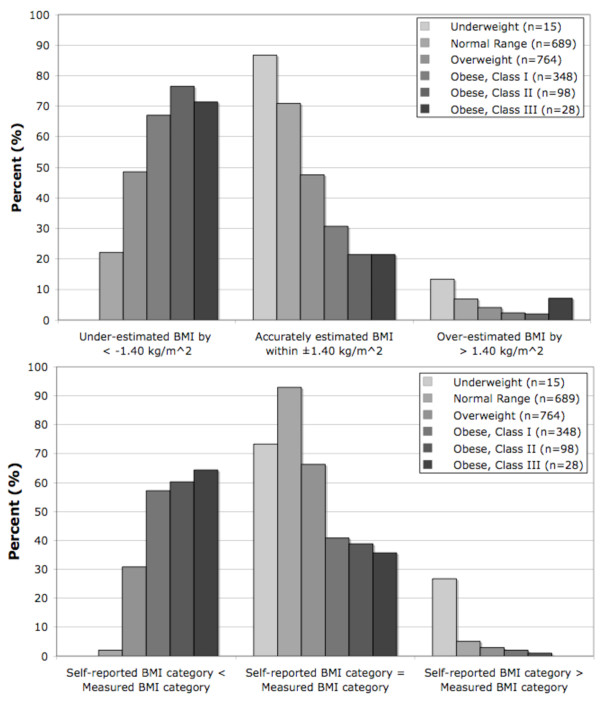
**Proportion of subjects with self-report-based BMI beyond or within ±1.40 kg/m^2 ^of measured BMI (A, top) and whose self-report-based and measured BMI resulted in concordant or discordant BMI category assignments (B, bottom)**.

**Figure 2:**
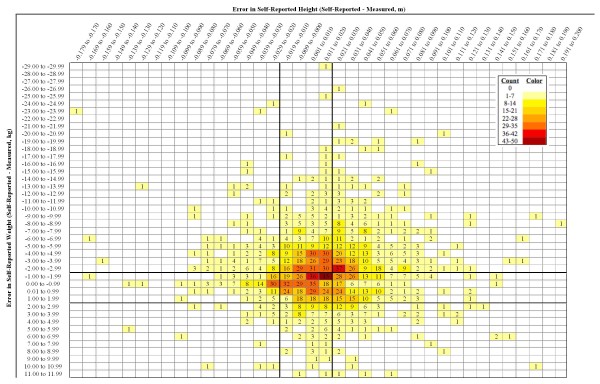
**Map of the frequencies of error in height and weight. Measured height (m) and weight (kg) were subtracted from their corresponding self-reported values to yield Error in Self-Reported Height (columns) and Error in Self-Reported Weight (rows)**. In both cases, negative values indicate that the self-reported value was less than the measured value. The degree of error was categorized in 1.0 cm or 1.0 kg intervals, and the frequency of error within each interval was determined. Solid lines indicate the cut-off values for accurately reported height (-0.02 m to +0.02 m) and accurately reported weight (-2.0 kg to +2.0 kg) to take into account technical errors of measurement and short-term fluctuations in weight and height. These lines form nine regions that correspond to the nine error pattern categories listed in Table 1. Counts are shown in each cell. Blank cells represent a count of zero, and each progressively darker shade represents the next seven-count level.

Measured weight (kg) and height (cm) were subtracted from their corresponding self-reported values to determine errors in self-reported weight and height. Degrees of error were categorized in 1.0 cm or 1.0 kg intervals, and the frequency of error within each interval was determined and plotted in a frequency map to demonstrate the distribution of errors in the study population (Figure 1). Using the definitions of accurately reported, under-reported, and over-reported weight and height, we assigned subjects to one of nine possible types of error (visually demarcated in Figure 2, listed in Table 1). The number and proportion of subjects for each error type was determined. The difference between self-reported and measured BMI (BMI_D_), an estimate of the degree and direction of bias, was calculated for the total population undergoing analysis and for each error type. To express the relative contribution of each error type to BMI_D_, the following calculation was used:

**Table 1:** Patterns of error in self-reported weight and height and their relative contributions to bias in BMI determined from self-reported values.

	Total				Normal Range			
	
	N	%	**BMI**_**D**_	**Relative Contribution to BMI**_**D**_	N	%	**BMI**_**D**_	**Relative Contribution to BMI**_**D**_
TOTAL	1942	100.0	-1.34		689	100.0	-0.49	

Accurate Weight								

Accurate Height	389	20.0	-0.17	-2.5	194	28.2	-0.16	-9.2

Under-reported Height	77	4.0	1.06	3.2	43	6.2	1.00	12.7

Over-reported Height	321	16.6	-1.48	-18.3	110	16.0	-1.12	-36.5

Over-reported Weight								

Accurate Height	92	4.7	1.42	5.0	51	7.4	1.31	19.8

Under-reported Height	24	1.2	3.01	2.7	14	2.0	3.28	13.6

Over-reported Height	96	4.9	0.00	0.0	52	7.5	0.15	2.3

Under-reported Weight								

Accurate Height	418	21.5	-1.92	-30.8	113	16.4	-1.45	-48.5

Under-reported Height	103	5.3	-0.53	-2.1	36	5.2	-0.19	-2.0

Over-reported Height	422	21.7	-3.54	-57.3	76	11.0	-2.34	-52.7
								
	
	**Overweight**				**Obese, Classes I-III**			

	**N**	**%**	**BMI**_**D**_	**Relative Contribution to BMI**_**D**_	**N**	**%**	**BMI**_**D**_	**Relative Contribution to BMI**_**D**_

TOTAL	764	100.0	-1.33		474	100.0	-2.66	

Accurate Weight								

Accurate Height	147	19.2	-0.19	-2.7	46	9.7	-0.21	-0.8

Under-reported Height	29	3.8	1.16	3.3	3	0.6	0.96	0.2

Over-reported Height	149	19.5	-1.63	-23.9	56	11.8	-1.95	-8.7

Over-reported Weight								

Accurate Height	27	3.5	1.43	3.8	13	2.7	1.89	1.9

Under-reported Height	7	0.9	2.75	1.9	2	0.4	-	-

Over-reported Height	27	3.5	-0.22	-0.6	15	3.2	-0.23	-0.3

Under-reported Weight								

Accurate Height	184	24.1	-1.75	-31.7	121	25.5	-2.61	-25.0

Under-reported Height	31	4.1	-0.62	-1.9	35	7.4	-0.84	-2.3

Over-reported Height	163	21.3	-3.00	-48.1	183	38.6	-4.53	-65.7

BMI_D _= BMI_SR _- BMI_M _(calculated for each subject, reported as mean values in table).

Self-reported height and weight were considered to be accurate when within ± 2.0 kg of measured weight or ± 2.0 cm of measured height.

Normal Range, BMI 18.5-24.9 kg/m^2^; Overweight, BMI 25.0-29.9 kg/m^2^; Obese, Class I-III, BMI ≥ 30.0 kg/m^2^.

Relative Contribution to BMI_D _= [(N_ErrorType_*Mean_ErrorType_)/(N_total _*Mean_total_)]*100b, where b = -1 when BMI_D _is negative and b = 1 when BMI_D _is positive to account for the type's direction of bias.

where *b *= -1 when BMI_D _is negative or *b *= +1 when BMI_D _is positive for a given error type. The factor *b *accounts for the error type's direction of bias. These analyses were carried out in the total sample and the normal, overweight, and obese BMI ranges. Underweight subjects were not analyzed in this manner because the number of observations (n = 15) was not large enough for stratification by error type but were included in the total population analysis. Interval estimates and statistical comparisons were not performed because the error types are not fully stochastic.

### Identification of factors associated with BMI bias and misclassification

Pearson χ^2 ^tests were used to compare the proportions of subjects in covariate sub-categories according to the definitions for under-estimated, accurately estimated, or over-estimated BMI (Table 2). Similar comparisons were performed for subjects whose self-report-based and measured BMI categories were negatively discordant (BMI_SR _category < BMI_M _category), concordant (BMI_SR _= BMI_M_), or positively discordant (BMI_SR _> BMI_M_) (not shown). Univariate and multivariate logistic regression were used to determine the associations (odds ratios [OR] and 99% confidence intervals [99%CI]) of self-described weight status or attempted weight management with the following binary outcomes: (1) under-estimated *vs *accurately estimated BMI, (2) over-estimated *vs *accurately estimated BMI, (3) negative discordance *vs *concordance, or (4) positive discordance *vs *concordance. Associations were also determined for under- or over-reported weight and height.

**Table 2:** Association of various factors and estimating BMI beyond or within ±1.40 kg/m^2^.

**Self-reported BMI**:	Under-Estimated		Correctly Estimated		Over-Estimated		
**BMI**_**D (self-reported - measured)**_:	**< -1.40 kg/m**^**2**^		**Within ±1.40 kg/m**^**2**^		**> 1.40 kg/m**^**2**^		
	N = 852 (43.8%)		N = 1000 (51.4%)		N = 93 (4.8%)		
	
	N	%	N	%	N	%	P value
BMI Category §							

Underweight	0	0.0	13	1.3	2	2.2	

Normal Weight	153	18.0	490	49.0	48	51.6	

Overweight	371	43.5	363	36.3	31	33.3	

Obese, Class I	233	27.3	107	10.7	8	8.6	

Obese, Class II	75	8.8	21	2.1	2	2.2	

Obese, Class III	20	2.3	6	0.6	2	2.2	< 0.001

Sex							

Male	366	43.0	449	44.9	47	43.0	

Female	486	57.0	551	55.1	46	57.0	0.327

Age							

18-29 y	90	10.6	184	18.4	16	17.2	

30-44 y	202	23.7	329	32.9	34	36.6	

45-64 y	331	38.8	366	36.6	29	31.2	

65+ y	229	26.9	121	12.1	14	15.1	< 0.001

Social class §§							

SC 1-2	334	37.6	382	39.8	26	26.8	

SC 3-4	366	41.2	372	38.8	40	41.2	

SC 5-6	119	13.4	134	14.0	20	20.6	

Unclassified	70	7.9	71	7.4	11	11.3	0.134

Current smoking status							

Former smoker	238	27.9	227	22.7	15	16.1	

Non-smoker	431	50.6	479	47.9	43	46.2	

Smoker	183	21.5	294	29.4	35	37.6	< 0.001

Alcohol consumption							

Never	183	21.6	137	13.7	23	25.0	

Monthly or less	145	17.1	150	15.0	25	27.2	

3-4 times/month	198	23.3	268	26.8	19	20.7	

2-3 times/week	235	27.7	338	33.8	18	19.6	

4 or more times/week	88	10.4	107	10.7	7	7.6	< 0.001

Marital status							

Single (never married)	185	21.8	244	24.5	28	30.1	

Married/Cohabiting	538	63.3	641	64.3	53	57.0	

Divorced/Separated/Widowed	127	14.9	112	11.2	12	12.9	0.066

Education							

Some/Completed Primary School	138	16.2	113	11.3	18	19.4	

Some/Completed Secondary School	387	45.4	423	42.3	48	51.6	

Diploma/Certificate	156	18.3	222	22.2	11	11.8	

Bachelors/Postgraduate Degree	171	20.1	242	24.2	16	17.2	0.001

Ethnicity							

White	826	98.2	971	97.9	90	96.8	

Black	5	0.6	4	0.4	1	1.1	

Asian	3	0.4	8	0.8	2	2.2	

Other	7	0.8	9	0.9	0	0.0	0.392

Physical Activity							

High IPAQ Score	221	25.9	214	21.4	25	26.9	

Medium IPAQ Score	425	49.9	514	51.4	47	50.5	

Low IPAQ Score	206	24.2	272	27.2	21	22.6	0.156

Self-described weight §§§							

About the right weight	419	49.6	607	60.8	48	51.6	

Too heavy	377	44.6	324	32.5	35	37.6	

Too light	22	2.6	42	4.2	3	3.2	

Not sure	27	3.2	25	2.5	7	7.5	<0.001

Attempting to manage weight							

No	406	47.7	540	54.0	49	52.7	

Lose weight	320	37.6	294	29.4	32	34.4	

Maintain weight	118	13.8	157	15.7	12	12.9	

Gain weight	8	0.9	9	0.9	0	0.0	0.020

BMI, body mass index; SC, social class; IPAQ, International Physical Activity Questionnaire.

§ BMI categories defined as: Underweight (<18.5 kg/m^2^), Normal Range (18.5-24.9 kg/m^2^), Overweight (25.0-29.9 kg/m^2^), Class I Obese (30.0-34.9 kg/m^2^), Class II Obese (35.0-39.9 kg/m2), and Class III Obese (40.0 + kg/m2).

§§ Household social class defined according occupation type, as per methods in ref. [23].

§§§ Self-described weight taking into account one's age and height.

All comparisons were made using Pearson chi-squared tests.

To determine self-described weight, subjects were asked: "Given your age and height, would you say that you are about the right weight, too heavy, too light, or not sure?" Attempted weight management was determined by asking subjects: "Are you actively trying to manage your weight?" If a respondent answered affirmatively, they were asked: "Is it to lose, gain, or maintain weight?" In a first analysis (Table 3, Model 1), associations were adjusted for age, sex, social class, ethnicity, marital status, highest level of education, physical activity level, current smoking status, and alcohol consumption. A subsequent analysis (Table 3, Model 2) included the same covariates as in Model 1 with BMI category as an additional covariate. A final model (Table 4) was constructed with all covariates, including both self-described weight and attempted weight management. All analyses were conducted using PASW Statistics v18.0 (Macintosh). Significance was set at P ≤ 0.01.

**Table 3:** Adjusted odds ratios (99% CIs) of self-described weight or attempted weight management and mis-estimation of BMI and discordance between self-reported and measured BMI categories

	**BMI**_**SR **_**Under-Estimated by < -1.40 kg/m**^**2**^		**BMI**_**SR **_**Category < BMI**_**M **_**Category**	
		
	Model 1^	Model 2^^	Model 1^	Model 2^^
Self-described weight §				

About the right weight	1.00	1.00	1.00	1.00

Too heavy	1.63 (1.24 to 2.14)***	0.46 (0.32 to 0.67)***	1.78 (1.33 to 2.39)***	0.21 (0.13 to 0.33)***

Too light	0.78 (0.38 to 1.61)	1.93 (0.87 to 4.28)^T^	0.91 (0.40 to 2.12)	22.3 (5.33 to 93.1)***

Not sure	1.51 (0.70 to 3.27)	0.89 (0.34 to 1.89)	1.68 (0.75 to 3.78)	0.53 (0.20 to 1.45)

Attempting to manage weight				

No	1.00	1.00	1.00	1.00

Lose weight	1.45 (1.09 to 1.93)***	0.59 (0.42 to 0.84)***	1.33 (0.97 to 1.81)^T^	0.28 (0.18 to 0.42)***

Maintain weight	0.97 (0.67 to 1.42)	0.91 (0.61 to 1.36)	0.90 (0.49 to 1.37)	0.82 (0.50 to 1.36)

Gain weight	1.18 (0.31 to 4.51)	2.24 (0.57 to 8.86)	0.98 (0.21 to 4.63)	6.88 (0.84 to 56.5)^T^

	**BMI**_**SR **_**Over-Estimated by > 1.40 kg/m**^**2**^		**BMI**_**SR **_**Category > BMI**_**M **_**Category**	
		
	**Model 1^**	**Model 2^^**	**Model 1^**	**Model 2^^**

Self-described weight §				

About the right weight	1.00	1.00	1.00	1.00

Too heavy	1.44 (0.76 to 2.72)	2.54 (1.10 to 4.82)**	1.49 (0.74 to 3.01)	3.87 (1.45 to 10.1)***

Too light	0.81 (0.16 to 4.13)	0.57 (0.10 to 3.39)	1.41 (0.27 to 7.27)	0.43 (0.06 to 3.33)

Not sure	3.02 (0.86 to 10.5)^T^	3.88 (1.06 to 14.1)**	2.15 (0.47 to 9.72)	3.37 (0.70 to 16.3)^T^

Attempting to manage weight				

No	1.00	1.00	1.00	1.00

Lose weight	1.43 (0.74 to 2.78)	1.99 (0.92 to 4.31)^T^	1.98 (0.97 to 4.03)^T^	4.48 (1.85 to 10.8)***

Maintain weight	0.99 (0.41 to 2.41)	1.02 (0.43 to 2.51)	0.77 (0.23 to 2.52)	1.01 (0.30 to 3.40)

Gain weight	-	-	1.93 (0.12 to 32.0)	1.94 (0.11 to 32.8)

All values reported above are adjusted OR (99% CI).

BMI_SR_, BMI based on self-reported weight and height; BMI_M_, BMI based on measured weight and height.

§ Self-described weight taking into account age and height.

^ Model 1: Multivariate logistic regression with adjustment for sex, age, ethnicity, current smoking status, alcohol consumption, physical acitvity level, level of education, and marital status.

^^ Model 2: Same as previous model but with BMI category as an additional covariate

^T ^P < 0.05, ** P < 0.01, *** P < 0.001.

**Table 4:** Associations of several variables in the final model with mis-estimation of BMI or discordance between self-reported and measured BMI categories.

	**BMI**_**SR **_**Under-** Estimated **by < -1.40 kg/m**^**2**^	**BMI**_**SR **_**Cat. <** **BMI**_**M **_**Cat**.	**BMI**_**SR **_**Over-** Estimated **by > 1.40 kg/m**^**2**^	**BMI**_**SR **_**Cat. >** **BMI**_**M **_**Cat**.
Sex				

Male	1.00	1.00	1.00	1.00

Female	1.76 (1.29 to 2.40)***	2.27 (1.54 to 3.33)***	0.51 (0.26 to 0.99)**	0.46 (0.21 to 1.01)^T^

Age				

18 to 29 years	1.00	1.00	1.00	1.00

30 to 44 years	1.15 (0.70 to 1.89)	0.69 (0.35 to 1.36)	1.43 (0.54 to 3.74)	2.12 (0.65 to 6.85)

45 to 64 years	1.48 (0.87 to 2.42)	0.90 (0.45 to 1.78)	1.22 (0.43 to 3.50)	1.69 (0.46 to 6.17)

65+ years	2.99 (1.65 to 5.41)***	1.20 (0.56 to 2.55)	1.28 (0.35 to 4.68)	1.80 (0.40 to 8.06)

BMI Category §				

Underweight	-	-	1.62 (0.16 to 16.47)	22.45 (2.82 to 179)***

Normal Range	1.00	1.00	1.00	1.00

Overweight	4.99 (3.42 to 7.29)***	112 (36.7 to 344)***	0.45 (0.20 to 1.03)^T^	0.24 (0.09 to 0.66)***

Obese	18.0 (10.7 to 30.4)***	1016 (300 to 3440)***	0.29 (0.09 to 0.91)**	0.12 (0.03 to 0.47)***

Marital Status				

Single (never married)	1.00	1.00	1.00	1.00

Married/Cohabiting	0.66 (0.45 to 0.97)**	0.60 (0.55 to 1.48)	0.75 (0.35 to 1.60)	0.82 (0.34 to 1.96)

Divorced/Separated/Widowed	0.64 (0.37 to 1.10)^T^	0.85 (0.44 to 1.62)	0.91 (0.30 to 2.74)	0.56 (0.14 to 2.25)

Self-described weight §§				

About the right weight	1.00	1.00	1.00	1.00

Too heavy	0.51 (0.34 to 0.76)***	0.28 (0.18 to 0.49)***	2.03 (0.80 to 5.14)^T^	2.20 (0.77 to 6.26)

Too light	1.76 (0.77 to 4.01)	19.8 (4.50 to 87.4)***	0.60 (0.10 to 3.54)	0.40 (0.05 to 3.05)

Not sure	0.81 (0.34 to 1.92)	0.60 (0.22 to 1.64)	3.77 (1.03 to 13.78)**	3.36 (0.68 to 16.5)

Attempting to manage weight				

No	1.00	1.00	1.00	1.00

Lose weight	0.74 (0.51 to 1.07)^T^	0.39 (0.25 to 0.60)***	1.55 (0.65 to 3.68)	3.50 (1.33 to 9.20)***

Maintain weight	0.88 (0.58 to 1.31)	0.73 (0.43 to 1.22)	1.02 (0.41 to 2.53)	0.97 (0.29 to 3.30)

Gain weight	1.79 (0.43 to 7.49)	2.19 (0.32 to 15.2)	-	2.52 (0.14 to 45.8)

All values reported are adjusted OR (99% CI).

BMI_SR_, BMI based on self-reported weight and height; BMI_M_, BMI based on measured weight and height; Cat., category.

§ BMI categories defined as: Underweight (<18.5 kg/m^2^), Normal Range (18.5-24.9 kg/m^2^), Overweight (25.0-29.9 kg/m^2^), and Obese (30.0+ kg/m^2^).

§§ Self-described weight taking into account one's age and height.

Multivariate logistic regression with the following covariates: sex, age, BMI category, ethnicity, current smoking status, alcohol consumption, physical acitvity level, level of education, marital status, self-described weight, weight management attempt.

^T ^P < 0.05, ** P < 0.01, *** P < 0.001.

### Ethics and Funding

SLÁN 2007 was approved by the Research Ethics Committee of the Royal College of Surgeons Ireland and was funded by the Department of Health and Children in Ireland.

## Results

### Self-reported weight, height, and BMI bias

As BMI_M _category increased, the prevalence of under-estimated BMI_SR _and of negative discordance between BMI_SR _and BMI_M _categories increased (Figure 1A and 1B, respectively, both P < 0.001). Opposite relationships were observed for the prevalence of over-estimated BMI_SR _and positive discordance, where in both cases the prevalence decreased with increasing BMI_M _category (Figure 1A and 1B). Underweight subjects had the highest prevalence of accurately estimated BMI_SR _(87%), with none of them having under-estimated BMI_SR_. Normal range subjects often had under-estimated BMI (22%), but they had the highest prevalence of concordance (93%), and only 2% had negatively discordant BMI categories. Obese subjects were the least likely to have accurate BMI_SR _estimates and to have concordant BMI categories.

To determine whether the trend for negative bias in BMI_SR _was due mainly to misreporting of weight or height, we assigned subjects to one of nine error types that correspond to the nine regions depicted in Figure 2 and listed in Table 1. Figure 2 visualizes the distribution of errors in self-reported weight and height and suggests a trend towards under-reported weight and over-reported height. Table 1 indicates that the degree of under-estimation in BMI_SR _increases as weight category increases. The overall BMI bias was -1.34 kg/m^2 ^and was -0.49, -1.33, and -2.66 kg/m^2 ^in the normal, overweight, and obese BMI ranges, respectively. Underweight subjects (n = 15) were excluded from this analysis because numbers in each group were too small for meaningful analysis. Although subjects in the normal BMI range exhibited a weak negative bias (-0.49 kg/m^2^), this degree of bias remained well within the acceptably accurate range of -1.40 to +1.40 kg/m^2^. The error types that contributed the most to bias in the total study population and in each of the BMI categories analyzed were consistently, in decreasing order of influence: (1) under-reported weight combined with over-reported height, (2) under-reported weight with accurately reported height, and (3) accurately reported weight with over-reported height.

### Identification of factors associated with BMI bias and misclassification

The proportions of subjects for each covariate category used in logistic regression analyses are shown in Table 2. Multivariate logistic regression without adjustment for measured BMI category (Table 3, Model 1) suggested that describing oneself as too heavy and actively attempting to lose weight were significantly associated with a greater likelihood of under-estimating BMI_SR _and exhibiting negative discordance. These covariates were also associated with an increased likelihood of under-reporting weight (OR 1.4, 99%CI: 1.1-1.8 for both covariates, P < 0.01) but not of over-reporting height (data not otherwise shown).

However, when Model 1 was further adjusted for measured BMI category, the associations described above were reversed (Table 3, Model 2). Specifically, in Model 2, describing oneself as too heavy and attempting to lose weight were, within an given BMI category, significantly associated with a lower likelihood of under-estimating BMI_SR _and exhibiting negative discordance within each BMI category. Describing oneself as too heavy was also associated with a lower likelihood of under-reporting weight (OR 0.5, 99%CI: 0.3-0.7, P < 0.001) and over-reporting height (OR 0.7, 99%CI: 0.6-1.0, P = 0.007). Similarly, attempting to lose weight was associated with a lower likelihood of under-reporting weight (OR 0.7, 99%CI: 0.5-1.0, P = 0.011) and over-reporting height (OR 0.7, 99%CI: 0.5-1.0, P = 0.008) (data not otherwise shown).

The associations described above in Model 2 for describing oneself as too heavy held when both covariates -- self-described body weight and attempting weight loss -- were entered simultaneously (Table 4). Attempting to lose weight was partially confounded by self-described weight. Four additional factors emerged as being significantly associated with BMI mis-estimation: sex, age, measured BMI category, and marital status. The strongest factor associated with all outcomes shown in Table 4 was BMI category.

## Discussion

In the present study, we developed a new methodological approach for the examination of bias in self-report-based BMI in any given sample of any given population. Based on this method, a consistent pattern of weight and height misreporting biases emerged. Specifically, in the total population and in the BMI categories analyzed, the combinations of misreporting biases that contributed the most to overall BMI mis-estimation were consistently, in decreasing order of influence: (1) under-reported weight combined with over-reported height, (2) under-reported weight with accurately reported height, and (3) accurately reported weight with over-reported height. Further examination of patterns of bias in BMI estimation revealed that subjects in the normal BMI range exhibited a slight negative bias overall but that the degree of bias was well within the acceptably accurate range. As BMI category increases beyond the normal range, both the prevalence and magnitude of self-report-based BMI mis-estimation increase. These latter findings regarding BMI categories are consistent with a large number of previous studies on weight and height misreporting biases in adults, suggesting that the method described herein has good reliability [2,5,6,8,10-13,15,16,18-20].

It is widely believed that research participants misreport anthropometric characteristics to portray a more socially desirable weight and height. This view has been echoed in the literature [16,24] despite limited evidence [4,17,18]. We provide evidence that challenges the role of social desirability as an explanation for misreported weight and height. Within any given BMI category, it appears that those who describe themselves as too heavy are less likely to under-report their weight, over-report their height, to have under-estimated BMI, and to be misclassified in a lower BMI category. Conversely, considering oneself to be too heavy increases the likelihood of over-reporting weight and having over-estimated BMI. These findings are consistent with those reported by Gil and Mora (2010) and by Villanueva (2001) [4,18]. Thus, although overweight and obese participants tend to under-report their weight, it seems that social norms concerning weight tend to reduce rather than exacerbate misreporting bias of this parameter.

A plausible explanation of this finding is that persons who describe themselves as too heavy are more weight-conscious. They may measure their weight more frequently and therefore report their anthropomorphic characteristics more accurately. We are unaware of data demonstrating a link between perceived weight status and weight consciousness or weighing frequency; however, in the present study those who were attempting to lose weight -- a behavior that is associated with greater self-weighing frequency [25,26] -- were, within any given BMI category, less likely to under-report their weight or to have under-estimated BMI. Rather, they were more likely to over-report their weight or to have over-estimated BMI. Further research is warranted to understand how social desirability influences self-reported weight and height and to explain why the degree of self-report-based BMI mis-estimation increases with progressively higher BMI categories. Indeed, in our final model presented in Table 4, BMI category was by far the strongest factor associated with the mis-estimation of BMI. The reasons for this association remain elusive.

The present study has important research implications, as the described methods can be optimized for any given sample from any given population and may provide novel insights into the factors associated with weight and height misreporting biases and self-report-based BMI mis-estimation. A particular advantage of this methodology is that it accounts for normal short-term fluctuations in weight and height and for technical errors of measurement, both of which may lead to apparent rather than true misreporting bias. Factors that may influence short-term weight and height variation may include food consumption (i.e., fasted vs post-prandial measurements), shifts in water balance/volume status, and temporal postural changes. Other factors influencing self-report accuracy may include reporting weight or height using a US standard scale rather than a metric one and reporting correct weight or height measurements taken weeks, months, or years earlier. Collectively, these factors may explain differences between self-reported and measured values, even in the absence of any intentional or unintentional misreporting.

A major weakness of this study is its cross-sectional design, which limits any causal inferences from our logistic regression analyses. In addition, the study population was mostly white, thereby precluding the study of ethnicity, a factor that some studies have shown to be associated with weight and height misreporting biases [4,17,27,28]. Another weakness is that some combinations of misreported weight and height lead to accurate BMI estimates. However, instances of accurate BMI estimates in the context of misreported weight and height were relatively uncommon, and the expected effect would be to bias logistic regression analyses towards the null hypothesis. Future studies utilizing the methodology reported herein should consider these limitations.

## Conclusions

A new methodological approach was developed for the examination of weight and height misreporting as well as BMI mis-estimation. This approach is useful for the exploration of patterns of such biases and for the analysis of factors potentially associated with misreporting bias. Using this new approach, we demonstrate that although BMI category is seemingly the most important factor associated with the misreporting of weight and height data, social norms concerning body weight appear to counteract such biases.

## Competing interests

The authors declare that they have no competing interests.

## Authors' contributions

JRB conceived of and designed the study, analyzed the data, interpreted the results, and drafted the manuscript. IJP obtained funding for SLÁN 2007, participated in data acquisition, interpreted the results, and reviewed the manuscript. JVDB contributed to the study design, interpreted the results, and drafted the manuscript. All authors approved the final manuscript.

## Pre-publication history

The pre-publication history for this paper can be accessed here:

http://www.biomedcentral.com/1471-2458/11/331/prepub

## Supplementary Material

Additional file 1 **Method of BMI error simulation to establish a BMI accuracy definition**. This file describes how to simulate the effects of various combinations of weight and height misreporting on BMI estimation in any given sample. This method is used to define the limits of acceptably accurate BMI estimation. A supplementary figure showing the application of this method in the present study is provided. Click here for file
