# Supplementary material for: Challenging the role of social norms regarding body weight as an explanation for weight, height, and BMI misreporting biases: Development and application of a new approach to examining misreporting and misclassification bias in surveys
Source: BMC Public Health. 2011 May 18;11:331. doi: 10.1186/1471-2458-11-331 (PMC3121629; doi:10.1186/1471-2458-11-331)
Supplement: Additional file 1 — Method of BMI error simulation to establish a BMI accuracy definition. This file describes how to simulate the effects of various combinations of weight and height misreporting on BMI estimation in any given sample. This method is used to define the limits of acceptably accurate BMI estimation. A supplementary figure showing the application of this method in the present study is provided. [file 1471-2458-11-331-S1.DOC]

ADDITIONAL FILE 1

**Method of BMI error simulation to establish a BMI accuracy definition**

A simulation of the effects of error in self-reported weight and height on BMI estimation was completed in order to form BMI accuracy definitions. Simulated errors in weight and height were defined by the following equations:

and

where self-reported values are simulated and measured values represent the average measured weight and height in a study population. Simulated errors in weight (0.5 kg intervals) and height (1.0 cm intervals) form rows and columns, and each simulated pair defines a cell within which the following equation was entered to calculate the effects of varying degrees of error in weight and height on BMI bias (BMI_Bias_):

Although this equation reduces to the difference between a simulated self-report-based BMI and the average measured BMI for a study population, the unreduced equation above is more readily usable in Microsoft Excel. By using a study sample’s average measured weight and height in the simulation, one can calibrate the BMI accuracy definition to that sample. The average measured weight in this study population was 76.2 kg, and the average measured height was 1.67 m (corresponds to a BMI = 23.7 kg/m^2^). Figure S1 below shows the values of BMI_Bias_ for each pair of simulated errors in weight and height. A value of zero represents a lack of bias, negative values represent negative bias, and positive values represent positive bias.

The height and weight accuracy definitions described in the Methodology section of the main text were used to identify the region in Figure S1 that should be considered acceptably accurate (shaded in dark green). All of the values in the dark green area had acceptably accurate self-reported weight and height as well as a BMI_Bias_ within ± 1.40 kg/m^2^; thus, BMI bias within this range was considered to be acceptably accurate. The area shaded in light green also represents simulations with an acceptably accurate degree of BMI bias but with inaccurately reported self-reported weight and/or height. Figure 2 and Table 1 in the main text demonstrate that these latter scenarios are relatively uncommon. Values < -1.40 kg/m^2^ were considered to be under-estimates (shaded in blue), and those > +1.40 kg/m^2^ were considered to be over-estimates (shaded in yellow).

**SUPPLEMENTARY FIGURE LEGEND**

**Figure S1.** Simulation of the effects of varying degrees of error in self-reported weight and height on BMI bias. Errors in weight and height were defined as the difference between a simulated value and the average measurement for the study population. Each pair defines a cell within which the effects of the corresponding errors in weight and height on BMI bias are reported. The height and weight accuracy definitions described in the Methodology section of the main text were used to identify errors in BMI that should be considered acceptably accurate (region shaded with dark green). All of the values in the dark green area had acceptably accurate self-reported weight and height as well as a BMI_Bias_ within ± 1.40 kg/m^2^; thus, BMI bias within this range was considered to be acceptably accurate. The area shaded in light green also represents simulations with an acceptably accurate degree of BMI bias but with inaccurately reported self-reported weight and/or height. Values < -1.40 kg/m^2^ were considered to be under-estimates (shaded in blue), and those > +1.40 kg/m^2^ were considered to be over-estimates (shaded in yellow).


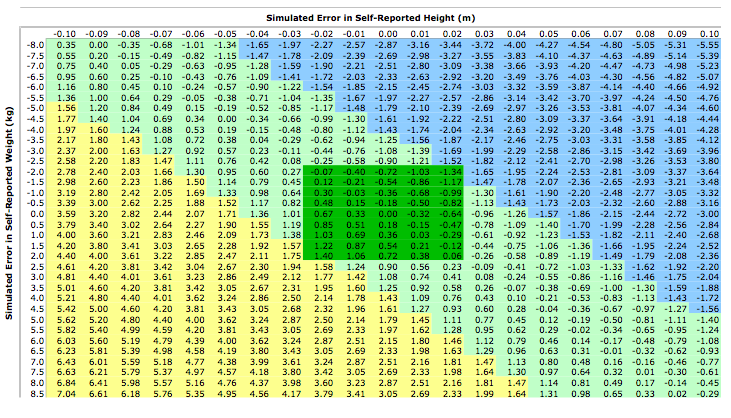
**Figure S1**
